# Glial Factors Regulating White Matter Development and Pathologies of the Cerebellum

**DOI:** 10.1007/s11064-020-02961-z

**Published:** 2020-01-23

**Authors:** Miren Revuelta, Till Scheuer, Li-Jin Chew, Thomas Schmitz

**Affiliations:** 1 Department for Neonatology, Charité University Medical Center, Augustenburger Platz 1, Mittelallee 9, 3353 Berlin, Germany; 2 Cellular Oncology Group, Biodonostia Health Research Institute, San Sebastian, Spain; 3 Center for Neuroscience Research, Children’s Research Institute, Children’s National Medical Center, Washington, DC USA

**Keywords:** Cerebellum, Glia, Development, Pathologies, Therapy

## Abstract

The cerebellum is a brain region that undergoes extremely dynamic growth during perinatal and postnatal development which is regulated by the proper interaction between glial cells and neurons with a complex concert of growth factors, chemokines, cytokines, neurotransmitters and transcriptions factors. The relevance of cerebellar functions for not only motor performance but also for cognition, emotion, memory and attention is increasingly being recognized and acknowledged. Since perturbed circuitry of cerebro-cerebellar trajectories can play a role in many central nervous system pathologies and thereby contribute to neurological symptoms in distinct neurodevelopmental and neurodegenerative diseases, is it the aim with this mini-review to highlight the pathways of glia–glia interplay being involved. The designs of future treatment strategies may hence be targeted to molecular pathways also playing a role in development and disease of the cerebellum.

## Introduction

The involvement of the cerebellum in higher processes of cognition and emotion [1, 2] and its relevance as a locus for a range of disorders and diseases make this simple yet elusive structure an important model in a number of fields. Cellular and anatomical dysfunction of the cerebellum has been associated with psychological disorders, such as autism, attention deficit, hyperactivity or schizophrenia [3–8]. In recent years, our understanding of some of the more familiar aspects of cerebellar growth, such as its territorial allocation and the origin of its various cell types, has undergone major recalibration. Furthermore, owing to its conserved circuitry across species, insights from comparative studies have contributed an increasingly rich picture of how this system develops. During fetal and postnatal development, the cerebellum undergoes dramatic morphological and structural changes, manifested as increased mass and a 30-fold increase of its surface area during the last trimester of pregnancy [9]. The regulation of its complex and dynamic development is driven by glial–glial and glia–neuron interactions, which produce a high variety of factors and molecules for interactive signal transmission [10]. Proliferation and migration of neural progenitor cells in the external granular layer (EGL) as well as the proliferation of immature glial cells are characteristic of late fetal and early postnatal development of the cerebellum. All of these processes are largely influenced or directed by the activity of the Purkinje cells [11–13] together with Bergmann Glia [14–16], by glia–glia [17–19], as well as by glia–neuron interactions [20–22], mainly through signaling via growth factors, chemokines and cytokines, transmitters and transcription factors. This review seeks to highlight shared mechanisms of glial cell regulation that are relevant for development and disease of the cerebellar white matter that may serve to design future strategies for protection.

## Glial Cell Function in Cerebellar White Matter Development

### Astrocytes

**Fig. 1:**
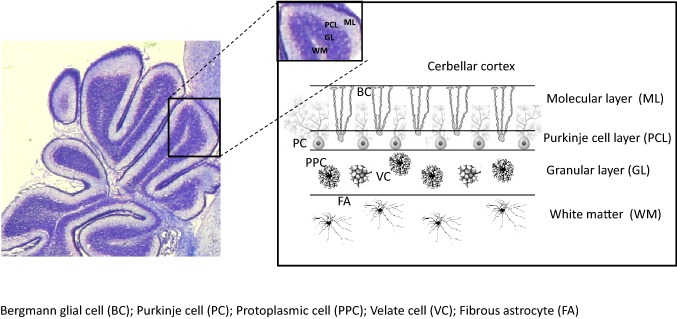
Cell types in the layers of cerebellar folia. *BC* Bergmann glial cell, *PC* Purkinje cell, *PPC* protoplasmic cell, *VC* velate cell, *FA* fibrous astrocyte

Astrocytes have a central role as supporting cells for neurons and oligodendroglia during brain development. Moreover, they represent a highly reactive cell population in numerous central nervous system (CNS) pathologies. Because of their importance in repair and recovery in neurological diseases, it has been suggested to use stem cell and progenitor cell derived astroglia for cell based therapy, e.g. in patients suffering from stroke, Alzheimer Disease, spinal cord disease, and others [23]. The structural and functional integrity of myelinated axons is critical for their reliable and efficient transmission of information. White matter injury has been associated with the development of many demyelinating diseases. Despite a variety of scientific advances aimed at promoting re-myelination, their benefit has proven at best to be marginal. Research suggests that the failure of the re-myelination process may be the result of an unfavorable microenvironment. Astrocytes are the most abundant and diverse type of glial cell in CNS which regulate cells of the oligodendrocytes lineage in diverse ways. As such, much attention has recently been drawn to astrocyte function in terms of white matter myelin repair. White matter astrocytes are different from those in gray matter in specific regards to development, morphology, location, protein expression and other supportive functions. During the process of demyelination and re-myelination, the functions of astrocytes are dynamic in that they are able to change functions in response to distinct stimuli or reactive pathways resulting in vastly different biologic effects. Their effects on oligodendrocytes and other cell types in the oligodendrocyte lineage include: serving as an energy supplier, a participant of immunological and inflammatory functions, a source of trophic factors and iron and a sustainer of homeostasis. As such, the ability to manipulate astrocyte function represents a novel therapeutic approach that can repair the damaged myelin that is known to occur in a variety of white matter-related disorders [23]. The properties of astroglia that are useful for neuroprotection are largely attributed to anti-oxidative properties, stabilization of glutamate homeostasis, and growth factor synthesis. In the cerebellum, astroglial cells are classified into four main groups based on morphology: fibrous astrocytes located in the white matter, stellate multipolar astrocytes or protoplasmic astrocytes located in the granular cell layer, and Bergmann’s glia (BG) located between the Purkinje cell layer and the molecular layer and that are specialized astrocytes derived from radial glia (Fig. 1). Developmental roles of astrocytes, particularly involving interactions with neurons, have been the subject of a recent review [24].

### Oligodendrocytes

**Fig. 2:**
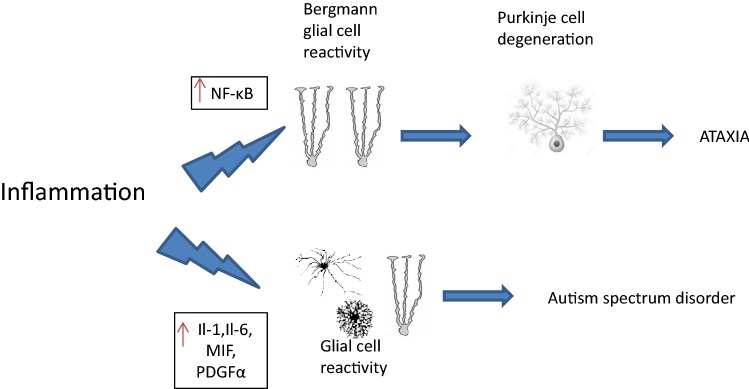
Pathways of glial–neuronal interaction in diseases triggered by inflammation, demonstrated by autism spectrum disorders and ataxia as examples

Oligodendrocytes development is strongly dependent on proper interaction with other types of glial cells, i.e. astroglia and microglia [17]. The establishment of the glial network represents an important step for healthy brain development [25]. Specifically, glial-derived growth factors regulate the survival, proliferation and maturation of glial cells, strongly influence the maturation and development of oligodendrocytes as well as myelination [26–28]. Cell culture experiments show that oligodendroglial cultures in astrocyte-conditioned medium survive and proliferate considerably longer than in microglial-conditioned medium [29]. In contrast, microglial-conditioned medium was reported to promote oligodendroglial differentiation and myelination due to its different pattern of cytokines and growth factors in the individual media [29]. The specific composition and the timing of certain cytokine and chemokine signaling appear essential for inducing either proliferation in order to expand the cellular pool during growth or maturation and network establishment (Fig. 2).

### Microglia

Microglia are the cells of the immune system in the CNS that make up about 10% of the total glial cells within the nervous tissue [30]. In the cerebellum, they distribute over white matter and the cortical layers during development. In the human embryo, colonization of the forebrain with microglia occurs at around 5 gestational weeks, while in rats this event takes place at embryonic day 11 [31]. Ramification as a process of microglial maturation occurs in the human mesencephalon between 11 and 22 gestational weeks, whereas in the cerebellum, the immature ameboid shape remains a predominant microglial phenotype [32]. Cerebellar microglia–Purkinje neuron interactions demonstrate properties distinct from cortical microglia [33]. Recent insight underline a role(s) of microglia for neurite growth, synaptic pruning, spinogenesis, and neuronal apoptosis during brain development [34–36]. Following experimental demyelination in rodents, oligodendrocyte precursor cells (OPCs) proliferate and differentiate into myelin-producing oligodendrocytes which effect robust remyelination. In contrast, remyelination in multiple sclerosis, the major human demyelinating disease, is generally limited and transient. Rodent OPCs have been well characterized in vitro and their response to growth factors documented. Several growth factors known to affect rodent OPCs were tested and found to have similar effects on human cells. PDGF, neurotrophin 3 (NT3), and glial growth factor 2 (GGF2) promoted proliferation, while insulin-like growth factor-1 (IGF-1), exerted a maturational effect [28]. Microglia can induce apoptosis of Purkinje neurons in vitro [37]. In the cerebellum, microglial functionality is needed for the elimination of excess climbing fibers and for proper GABA transmission by Purkinje cells [38].

Microglia has important functions in the maturation and development of oligodendrocytes. They secrete IGF1 and thus support the proliferation and maturation of OPCs [17, 28]. In addition, increased IGF1 stimulation protects immature oligodendroglia against damage triggered by inflammatory processes [29]. Pro-inflammatory, activated microglia interferes with the development of oligodendrocytes. Immature oligodendrocytes and OPCs are vulnerable to inflammatory processes induced by microglia. The survival of immature oligodendroglia and OPCs is reduced by activated microglia. In contrast, survival of mature OLs is enhanced by activated microglia and reduced apoptosis [39].

In the immature brain, exposure to IL1β can cause acute white matter injury [26] and lead to persistent hypomyelination [40]. Microglial contribution to white matter damage via pro-inflammatory responses is also described in models of inflammatory neonatal brain injury and in multiple sclerosis models [41, 42]. IL1β has also been demonstrated to interfere with transmission of GABA and of glutamate in Purkinje cells [43].

Like neurons, glial cells are also vulnerable to non-physiological glutamate concentrations. All three types of glial cells express different glutamate receptors and transporters. Oligodendrocytes are very sensitive to excessive activity of the glutamate signaling pathway. Microglia is stimulated at elevated glutamate concentrations, leading to the synthesis of inflammatory cytokines. Astrocytes are responsible for glutamate uptake in synaptic and non-synaptic areas and represent the most important regulators of glutamate homeostasis [44]. In addition, they produce 90% of the brain-derived lactate [45], which is an important source of energy for oligodendrocytes during myelination [46].

## Cerebellar Pathologies as a Result of Disrupted Glial–Neuronal Interaction

For brain development, the interaction between glial cells and neurons is essential. This is reflected in the secretion and degradation of neurotransmitters, stimulation by growth factors and also by cell–cell contact, all influencing proliferation, maturation, migration and survival of glial cells and neurons [11, 47–52]. In the developing and also in the adult brain, it has been described that the function of glial cells can influence and regulate neuronal activity [53].

In the development of neurons, astrocytes are assigned an important partner role. In addition to the maintenance of homeostasis by the uptake and breakdown of neurotransmitters [44] and the supply of nutrients to the neurons [54], they are crucially involved in the formation and maturation of synapses [55]. They also support the outgrowth of axons and dendrites as well as the migration of immature neurons [56]. This mutual interaction can be controlled by neurons through the release of growth factors and of neurotransmitters [57].

During development, Bergmann glia, Purkinje cells (PC) and granule cells contribute to the formation of the cerebellar cortex. An average of eight Bergmann glia are in close contact with a PC, thus promoting differentiation, synaptic training, and the transmission of neurotransmitters [58]. The maturation of Bergmann Glia is in turn influenced by PCs: the expression of SHH by Purkinje cells stimulates the maturation and differentiation of the Bergmann glia [59]. In addition, SHH influences the secretion of gliotransmitters by astrocytes [22] and thus indirectly influences the stimulation of other cell populations by astrocytes.

In agreement with this, ablation of astrocytes and Bergmann glia leads to malalignment of Purkinje cells, and moreover to diminished outgrowth of the dendrites and increased apoptosis of granule cells. *BDNF*, e.g., secreted by astrocytes, is difficult to diffuse over long distances, so local secretion is crucial [60]. Studies have shown that astroglia express both BDNF and the BDNF receptor [19]. BDNF production by Bergmann glia is directly involved in the migration of immature GCs from EGL into the IGL [61].

In addition to astrocytes, microglia also express BDNF during brain development [21]. Microglia of the cerebellum can modulate synaptic circuitry and synaptic activity between GCs and Purkinje cells through the secretion of BDNF [62]. Microglia may exert neuroprotective properties for cerebellar neurons, however, activation of microglia can also be toxic to immature and mature neurons [63].

It has been proposed that synergy between GABAergic synapses and astrocytic processes is limited to Bergmann glia in the cerebellum [64]. Indeed, microglia expresses the GABA B receptor. GABA has a modulating effect on microglia and can attenuate or block their activation with concurrent release of pro-inflammatory cytokines and phagocytic actions [65].

A fundamental and almost symbiotic co-existence of two distinct cell types of the brain can be seen in the intimate interaction between oligodendrocytes and neuronal axons. The formation of a myelin sheath around nerve fibers by oligodendrocytes is critical for an efficient and low-energy stimulus transmission [66]. Electrical transmission itself represents a key signal for oligodendroglia to initiate and enhance the wrapping of axon with myelin [67, 68]. In addition to myelin synthesis, oligodendroglia (OLs) have further influences on the axons of the neurons. It is assumed that OLs provide neurons with additional nutrients via their axons. Inhibition of nutrient transport by oligodendrocytes leads to the degradation of axons and neurons [69].

During development, the interaction of neurons and oligodendrocytes and their precursors plays an important role. Only through contact with an axon is the final maturation of the OLs initiated [66]. Oligodendrocytes express receptors for various neurotransmitters, such as the AMPA receptor [70], the NMDA receptor [71], and GABA A and the GABA B receptor [51, 72]. The blockade of the release of synaptic vesicles and neurotransmitters leads to impaired myelination [67]. In particular, stimulation with GABA is important for the development of OLs [50, 73]. The proliferation, maturation and migration of immature oligodendrocytes is regulated by GABA, in the first postnatal weeks, stimulation with GABA may be crucial for the development of OLs [74].

Decreased myelination has a major impact on the function and maturation of neurons. In a model for the ablation of oligodendrocytes with no myelination, there is a disrupted interaction in the cerebellum between Purkinje cells and the immature progenitors of the granule cells in the EGL. The reduction is also associated with an altered maturation and morphology of PC dendrites [75]. Hence, impairment of one factor relevant to neuron–glia crosstalk may in fact lead to dysregulation of multiple signaling pathways between neurons and glial cells, disrupting development of the cerebellum in multiple ways.

## Cerebellar Glial Cell Alterations in Diseases

There are many diseases in which glial changes in the cerebellum are involved, such as ataxia, leukoencephalopathy, autism and attention-deficit/hyperactivity disorder (ADHD), multiple sclerosis, as well as hypothyroidism which characteristically involve severe glial dysfunction (Table 1).

**Table 1:** Glial cells mechanism in cerebellar development and disease

Glial cells in the cerebellum	Mechanism in celebellar development	Mechanism in disease
Astrocytes	Secrete cytokines and growth factors → oligodendrocytes and myelin modulation Bergmann glia → provides a structure for cerebellar neuron migration and positioning	*Glial inflammation disorders* EAE → proinflammatory cytokine release (IL-1β) SCA1 → Bergmann glial cell reactivity through NF-Κβ *Neuron–glial interaction disorders* ADHD → increased GABA levels SCA7 → GLAST function interference, cause Purkinje cell excitotoxicity *Oxidative stress disorders* Neonatal ischemia → increased Ca^2+^ influx in Bergmann cells
Oligodendrocytes	Cerebella cytoarchitecture maintenance Oligodendrocytes–Neuron interaction maintains and forms Ranvier nodes and paranodal regions of Purkineje cell	*Oxidative stress disorders* Postnatal hiperoxia → oligodendroglial maldevelopment Postnatal hypoxia → hypomielinization and reduced oligodendroglial maturation
Microglia	Regulate neurite growth, synaptic pruning, spinogenesis, neuronal apoptosis and oligodendrocyte maturation and development	*Inflammation disorders* EAE → increased INFβ release SCA1 → inflammation (increase of TNFa) SCA3 → upregulation of (MMP-2, IL-1 and SDF1alpha)

### Glial Inflammation Disorders

When glia are activated, inflammation is amplified by the secretion or expression of inflammatory cytokines, chemokines or inducible nitric oxide synthases (iNOS) [76]. The molecules that are released after glial activation, can promote inflammation or exert anti-inflammatory properties. Astrocyte-specific changes analyzed by transcriptomics include decreased cholesterol biosynthesis and increased immune pathway gene expression [77]. Astrocyte cell endfeet contain aquoporin (AQP4) that contributes to regulating the junctional exchange of ions with blood vessels [78]. Among proinflammatory molecules, AQP4 has an important role in controlling brain edema as it is one of the most abundant water channels controlling the water influx in the brain parenchyma [79]. Among anti-inflammatory molecules, TGFβ, responsible of controlling neuroinflammation, is one of the cytokines that is upregulated after glial activation [80] as well as some neurotrophic factors that are release by astrocytes and microglia after an inflammation and are responsible of neuron protection [81].

#### Multiple Sclerosis (MS)

AQP4 is one of the most important proinflammatory molecule that is expressed in cerebellum and although its expression level is extremely low in the first postnatal week, it dramatically increases in the second week [82]. In progressive MS, cerebellar lesions frequently present as demyelination in white and gray matter regions [83–85]. Reactive astrocytes are a common feature of MS demyelinating lesions, with observed damage to astrocyte endfeet [86]. In an experimental autoimmune encephalomyelitis (EAE) model relevant to multiple sclerosis (MS), it was observed that the AQP4 increase in the cerebellum is associated with BBB disruption by decreased tight junction proteins, like occludins [87].

In this acute phase of EAE model, in, there is a glutamate-mediated synaptic excitability and neurotoxicity due to the astrocytic release of proinflammatory cytokine interleukin-1β (IL-1β) [88, 89]. This systemic cytokine exposure has been linked to hypomyelination and microglial activation in a perinatal inflammation model [90]. Hence, glial interleukin-1β may play a central role in microglial activation and glutamate excitotoxicity in inflammatory diseases of the cerebellum, too.

In a MOG-induced EAE model, increased release of INFβ by microglia induces demyelination, and increased density of IFNβ+ microglia are found around white matter lesions [91]. As a therapeutic agent, IFNβ represents a widely used treatment regimen for patients with relapsing–remitting MS (RRMS) [92] and shows treatment efficacy by reducing disease progression and also frequency of exacerbation. In animal experiments, induction of endogenous IFNβ by polyinosinic:polycytidylic acid [poly(I:C)] treatment diminished the severity of EAE, and genetic deletion of IFNβ or its receptor in contrast enhanced clinical score, with more extensive CNS inflammation and demyelination [93]. Treament with IFNβ also reduced axonal damage in a cerebellar slice culture assay with LPS stimulation [94].

#### Ataxia

One of the main conditions involving astroglial inflammation of the cerebellum is ataxia or lack of coordination. Ataxia is associated with many neurological conditions, such as stroke, brain tumor, multiple sclerosis, traumatic brain injury, toxicity, infection or congenital cerebellar defects [95]. In particular, spinocerebellar ataxia (SCA) is a group of hereditary ataxias that are characterized by degenerative changes in cerebellum. Mutations in many different genes are known to cause the different types of spinocerebellar ataxias (SCA) [96].

Among the Spinocereberllar ataxias, type 1 (SCA1) is the best known autosomal dominant neurodegenerative disease caused by the abnormal expansion of CAG repeats in the coding region of Ataxin 1 gene [97]. Cvetanovic et al. [98] described astrocytic and microglial activities as an underlying cause of SCA1 which is characterized by the loss of Purkinje neurons in the cerebellum. In that study, Cvetanovic et al. proposed that Bergmann glial cell reactivity signaling through NF-kB,, can be responsible for the pathogenesis of Purkinje cell during SCA1, because of their location and intimate interaction [99]. Furthermore Ferro et al. [97] found that the inhibition of NF-κB in microglia of SCA1 decreased the density of microglia and TNFα expression.

In spinocerebellar ataxia type 3 (SCA3), in which abnormal CAG repeats are localized in the coding region of a gene encoding ataxin-3, there is upregulation of matrix metalloproteinase 2 (MMP-2), interleukin-1 and the cytokine stromal cell-derived factor 1alpha (SDF1alpha) due to astroglial and microglial inflammation [100], causing abnormalities in the Purkinje cell. Recently, it has been suggested that antisense oligonucleotides (ASOs) may serve as a potential therapy technique for SCA3 [101].

#### Autism Spectrum Disorder

A psychiatric pattern that seems to be related to glial cell inflammation in the cerebellum is described in autism spectrum disorders (ASD), which begin during early childhood development and are influenced by genetic and environmental factors. The cerebellum has been described to be a brain region of particular relevance for ASD, and for some of the characteristical symptoms of the disorder. It has been suggested that cerebro-cerebellar connectivity is aberrant in ASD patients [102, 103]. Available research studies suggest that chronic neuroinflammation may represent a substantial pathogenic influence in the disease. Altered expression of proinflammatory cytokines and chemokines, such as IL-1, IL-6, macrophage migration inhibitory factor (MIF) and platelet derived growth factor (PDGF) has been demonstrated in ASD patients in the peripheral blood or in brain tissues [104]. The relevance of systemic inflammation for ASD symptoms is also revealed by successful treatment of children with diagnosis of ASD using autologous stem cell infusions, which resulted not only in impressive reduction of symptoms [105] but also in reduction of serum cytokine levels [106]. Dysregulated inflammatory activity in glial cells of the CNS, and specifically in the cerebellum, may therefore represent a therapeutic target in ASD.

### Neuron–Glia Interaction Disorders

The role of neuron–glia interaction in neurodegenerative disorders still remains unknown. The cerebellum, due to its simple anatomical organization and well-characterized circuitry, can be a useful tool to approach disorders of neuron–glial interactions [107].

*Attention-deficit/hyperactivity disorder (ADHD)* is a behavioral and developmental neurological disorder characterized by motor hyperactivity and loss of impulse control, combined with attention deficits and hampered academic performance [108]. A link to cerebellar pathologies has been revealed in clinical studies showing decreased cerebellar volume during in ADHD patients [109]. In G protein-coupled receptor kinase-Interacting protein-1 (GIP1) knockout mice, a genetically modified ADHD model, there is a decrease in GABA levels in astrocytes of the cerebellum that enhances the excitatory/inhibitory input ratio, leading to motor hyperactivity in ADHD. However the mechanism of GABA reduction is still unknown [110, 111].

*Spinocerebellar ataxia type 7 (SCA7)* is an autosomal dominant inherited neurodegenerative disorder with a polyglutamine (polyQ) expanded protein in the nuclear inclusions, and CAG trinucleotide repeats in the coding region of Ataxin-7 [112]. Indeed, it has been identified that polyQ expanded ataxin-7 interfered with the function of GLAST, a glia-specific glutamate transporter which is highly expressed in Bergmann glia, causing Purkinje cell excitotoxicity [106].

### Oxidative Stress in Cerebellar Glial Cells

Cerebellar damage in very immature infants can range from the subtle—generalized delay of tissue development and maturation in response to oxidative stress and/or systemic perinatal inflammation,—to severe—bleeding after rupture of the immature vessels, hence leading to focal lesions and parenchymal cysts as sequel. In a newborn rodent model, the great vulnerability of the immature cerebellum in response to oxidative stress has been characterized by maturational delay in oligodendroglial lineage cells, hypomyelination, and inflammatory changes in microglia [113].

#### Ischemia

After brain ischemia, as a response to inflammation, there is a generation of reactive oxygen species (ROS) in the neonatal and adult brain. Among the many ROS producers, the most important ones seem to be the NADPH oxidase (NOX) as the main superoxide producer [114], Xanthine oxidase (XO), that contributes to brain edema, and the intracellular enzymes such as COX lipoxygenases (LOXs), and cytochrome P450 that are involved in the arachidonic acid metabolism, a major superoxide source during ischemic stroke in the brain [76]. Moreover, the mitochondrial electron transporter chain is another important ROS source in the neonatal and adult brain. During reperfusion after ischemia, a massive increase of intracellular Ca2+ influx may be induced, and Ca2+ accumulation in the mitochondria can provoke free radical production, impairment in mitochondrial membrane permeability and inhibition of ATP production [115]. Particularly in the cerebellum, during oxygen glucose deprivation (OGD), anoxic depolarization of Purkinje cell in cerebellar slices invokes glutamate release from AMPA receptor activation. Indeed, this glutamate release has been proposed to be regulated by glial pH changes [116]. Moreover, after OGD, Bergmann glial cells, increased intracellular Ca2+ influx and membrane depolarization due to the increase of extracellular K+ concentration with the outflow of anions through DIDS sensitive channels [117].

#### Postnatal Hyperoxia

In utero*,* arterial oxygen tension is maintained at low levels but premature birth can provoke an increase in arterial oxygen tension upon exposure to the ex utero environment [118]. Scheuer et al. in 2015 found increased levels of nitrotyrosine in the cerebellar lysates correlated to cerebellar volume deficit, increases apoptosis in oligodendroglia precursor cells (OPCs) and a significant in vivo reduction of astroglial PDGFα, BDNF, FGF2 that may contribute to oligodendroglial maldevelopment. After hyperoxia, ultrastructure analysis by electron microscopy indicated thinning of the myelin sheath around the axon. In those experiments, markedly reduced PDGF-A expression was found in the cerebellum. The reduction of PDGF-A expression by high oxygen levels was confirmed in purified astrocyte cultures in vitro, suggesting the impairment of astroglia-oligodendroglia-crosstalk as a cause of cerebellar injury [118]. However, astroglial morphology and GFAP expression were not affected by hyperoxia. Consistent with delayed maturation of microglia in the cerebellum, most of the Iba1 microglia in the cerebellar white matter were of ameboid morphology in postnatal rats cerebella under control and hyperoxia conditions. There were otherwise no obvious hyperoxia-induced changes in morphology or antigen presentation in microglia in the cerebelli of hyperoxia animals.

There are certain compounds that can also induce oxidative stress in the cerebellum, such as Phytanic acid (3,7,11,15-tetramethylhexadecanoic acid, Phyt). Phyt is a chlorophyll derived acid that is obtained from daily products, such as milk, cheese or red meat. The accumulation of this fatty acid provokes many peroxisome disorders. Particularly in the cerebellum, it can induce histopathological abnormalities, including Purkinje cells alteration with a cellular loss and delayed dendrite development and astrogliosis due to the disruption of redox homeostasis. Indeed, in a mouse model of Phyt intracerebellar administration reactive nitrogen species were increased [119], indicating the potential risks to cerebellar integrity.

#### Postnatal Hypoxia

In a perinatal brain injury model, the application of chronic hypoxia within the first weeks of postnatal development leads to hypomyelination of the subcortical white matter [120]. Oligodendroglial damage has also been described in the cerebellum; altered development of the cerebellar white matter after chronic hypoxia has been described to be caused, at least partially, by the loss of GABAA receptor-mediated synaptic input to cerebellar OPCs, which enhances OPC proliferation and reduces oligodendroglial maturation and myelin synthesis [73].

## Targets for Potential Therapy

Brain diseases often involve inadequate homeostasis in neuronal and glial cells. In astroglia, pathogenic changes can be found in diverse processes e.g., glutamate uptake, neurotrophins, growth factors, transcription factors, anti-oxidative capacity, transmitters, as aforementioned. Consequently, these factors and pathways are offering treatment opportunities via prevention of toxicity or via activation of mechanisms of protection and repair.

### Inflammation Regulation

NF-κB is a key transcription factor implicated in neuroinflammation which may mediate events in cerebellar astrogliosis. Indeed, during inflammation NF-κB is activated and IKK is phosphorylated [121]. This activation produces neurotoxic and inflammatory molecules that lead to different diseases. With regard to SCA1, one of the main diseases related to astroglial inflammation in the cerebellum, it has been suggested that NF-κB signaling is stage dependent and the activity in SCA1 and the of NF-κB occurs only in the last stages of the SCA1 [121]. Moreover, Kim and co-workers [99] performed selective inhibition of NF-κB in astroglial cells, which in early stages has in fact increased motor deficits, higher Purkinje cell pathology and increased microglial density. With inhibition in late stages however, SCA1 motor deficits are ameliorated, accompanied by better rotarod performance and decreased microglial density. Interestingly, GFAP expression was decreased during the inhibition of NF-κB in early stages while it was increased in late stages, indicating that astroglial NF-κB pathway is beneficial during early, pre-syntomatic stage of the disease and it´s inhibition during late stage has also beneficial outcomes in SCA1 desease [121].

### Minocycline

Neuroprotective properties of this antibiotic have been demonstrated in different brain injury models, including hypoxia–ischemia [122–124] perinatal inflammation/infection [125] and hyperoxia [113]. The mechanisms by which minocycline exerts its benefits have largely been ascribed to inhibition of microglia. In the immature brain, inhibition of microglia may in fact perturb neuronal development and survival [126]. Toxic effects have been reported to vary with species, i.e. in mice, minocycline enhances brain injury caused by hypoxia–ischemia [127]. Extensive safety tests are therefore required. In an oxidative stress challenge, protection by minocycline coincided with attenuation of oxidative stress and of apoptotic cell death [113], which is supporting previous results on anti-oxidant and anti-apoptotic effects of this drug [128].

### Oxidative Stress Modulation

Glutamate neurotoxicity is directly associated with ROS production and consequently to oxidative stress [129, 130]. The *Amburana cearensis*, a species of the family of Fabaceae, has been observed to have antioxidant properties in the cerebellum that increase the levels of glutathione reductase and glutathione peroxidase enzyme. These control the intracellular signaling cascade of glutamate exitotoxicity that stimulates calcium influx and mitochondrial dysfunction, minimizing glial and neuronal cell death. In cerebellum astrocyte-derived cell culture, Amburana cearensis antioxidant compounds increase glutamine synthetase activity, which reduces glutamate neurotoxicity in astrocytes. Another compound important for redox balance in the cerebellum is the docosahexaenoic acid (DHA), the most abundant n-3 fatty acid in the brain derived from fish. DHA is essential for normal brain function and astrocytes are responsible for DHA synthesis [131, 132]. Indeed, it has been recently suggested that supplementation with DHA can be an effective treatment against spinocerebellar ataxia 38 (SCA38) a syndrome characterized by the mutation in the ELOVL5 gene that encodes an elongase enzyme responsible for very low chain fatty acids in the cerebellum [133].

### Growth Factors

The protection of cerebellar white matter development by minocycline was associated with improved PDGF-A expression in vivo and in astrocyte cultures in vitro, underlining a role for astroglial PDGF-A both in injury and protection in the cerebellum. Administration of PDGF-A intranasally after exposure to oxygen challenge moreover resulted in enhanced proliferation of oligodendroglial lineage cells in the cerebellar white matter [134], hence strengthening the view of growth factor synthesis as a target for protective treatment after postnatal insult.

In the chronic hypoxia model of white matter damage in the immature brain, overexpression of the human the receptor of epidermal growth factor (EGF) in oligodendroglial lineage cells after injury attenuates oligodendroglia cell death, increases the generation of new oligodendroglia from progenitors, and initiates recovery [135]. Moreover, intranasal administration of heparin-binding EGF during recovery after exposure to hypoxia enhanced OPC pool and oligodendroglial maturation, and also diminished ultrastructural pathologies and behavioural deficits. Hence, targeting the EGF receptor in oligodendrocyte progenitor cells during a certain time window is potentially beneficial for treatment of preterm infants with white matter damage. Nonetheless, these investigations were performed in the cerebrum/forebrain, a similar therapeutic effect of EGF administration on oligodendroglial maturation during postnatal development can be assumed to occur in the cerebellum, too.

### GABA Modulation

Balancing excitatory and inhibitory synaptic transmission is necessary for a proper brain function. Indeed, one of the main inhibitory neurotransmitter is the c-Aminobutyric acid (GABA) involved in neural tissue development. It has been suggested that mice treated with GABAA receptor antagonist mimics hypoxia effects, so the blockade of GABA uptake reduces NG2 progenitor cell numbers and increases the formation of mature oligodendrocyte [73, 136]. Recently Woo et al. [137] suggested that the manipulation of the levels of astrocytic tonic GABA in the cerebellum and in particular, in Bergmann glial cell, modulates neuronal excitability and synaptic transmission in the cerebellum. Moreover, the pharmacological inhibition of Bestrophin 1 (Best1), a protein that inhibit GABA release in Bergmann glial cells and the inhibition of mitochondrial enzyme monoamine oxidase B (MAOB), a protein in charge of GABA synthesis in astrocytes, causes an increased neuronal excitability in cerebellar granule cells, synaptic transmission and motor performance on the rotarod test. Conversely, increased astrocytic GABA release resulted in reduced motor activity, indicating that the astrocytes are a key component modulating GABA function and consequently modulating motor activity [137].

## Conclusions

The cerebellum is a brain region that is involved in many complex brain functions such as coordination, cognition, memory, emotion. In several neurodevelopmental and neurodegenerative diseases, damage of the cerebellum contributes to overall neurological symptoms. Given the fundamental role of glial cell types and glia–glia interactions for development, disease, and repair in the cerebellum, it is reasonable to target specific properties and functions of these cells for therapeutic purposes. For future investigations, growth factors like PDGFA and EGF, homeostasis of transmitters such as GABA and glutamate, various anti-oxidants and inflammatory modulators altogether represent a promising list of candidates that may serve for cerebellar protection.
